# Molecular mechanisms of cellular metabolic homeostasis in stem cells

**DOI:** 10.1038/s41368-023-00262-z

**Published:** 2023-12-01

**Authors:** Xiaoyu Li, Ou Jiang, Songlin Wang

**Affiliations:** 1 Salivary Gland Disease Center and Beijing Key Laboratory of Tooth Regeneration and Function Reconstruction, Beijing Laboratory of Oral Health and Beijing Stomatological Hospital, Capital Medical University, Beijing, China; 2 Department of Biochemistry and Molecular Biology, School of Basic Medical Sciences, Capital Medical University, Beijing, China; 3 Immunology Research Center for Oral and Systemic Health, Beijing Friendship Hospital, Capital Medical University, Beijing, China; 4 Laboratory for Oral and General Health Integration and Translation, Beijing Tiantan Hospital, Capital Medical University, Beijing, China; 5 Research Unit of Tooth Development and Regeneration, Chinese Academy of Medical Sciences, Beijing, China

**Keywords:** Stem cells, Cell biology

## Abstract

Many tissues and organ systems have intrinsic regeneration capabilities that are largely driven and maintained by tissue-resident stem cell populations. In recent years, growing evidence has demonstrated that cellular metabolic homeostasis plays a central role in mediating stem cell fate, tissue regeneration, and homeostasis. Thus, a thorough understanding of the mechanisms that regulate metabolic homeostasis in stem cells may contribute to our knowledge on how tissue homeostasis is maintained and provide novel insights for disease management. In this review, we summarize the known relationship between the regulation of metabolic homeostasis and molecular pathways in stem cells. We also discuss potential targets of metabolic homeostasis in disease therapy and describe the current limitations and future directions in the development of these novel therapeutic targets.

## Introduction

**Fig. 1:**
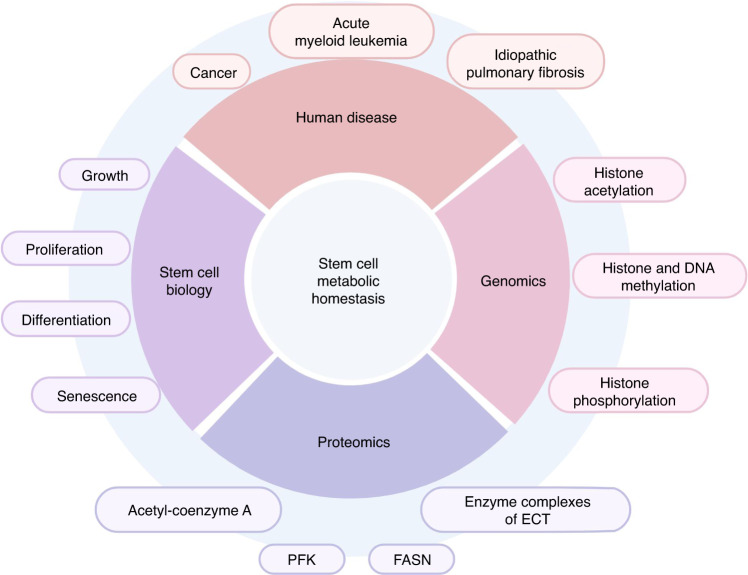
The significance of stem cell metabolic homeostasis. The metabolic homeostasis of stem cells plays an essential role in genomics, proteomics, cell biology, and the overall functioning of the organism. Dysregulation of the metabolic balance of stem cells will lead to serious adverse consequences. Created with BioRender. PFK phosphofructokinase, FASN fatty acid synthase

As primitive and unspecialized cells, stem cells have strong regenerative capacities, which are key to maintaining homeostasis of the mammalian internal environment and regeneration of tissues and organs. Stem cells have great potential for therapeutic development and clinical applications in fields such as disease treatment, tissue repair, and drug development. Cellular metabolism refers to a series of chemical reactions that occur in stem cells, including material transformation, energy conversion, and signal transduction. The metabolic state of cells is mainly a result of the maintenance of specific stem cell functions at different stages.^1^ However, new evidence suggests that cellular metabolism can also determine the biological functions of stem cells, thereby directly controlling their fate.^2,3^ Thus, a growing body of research supports the hypothesis that proper maintenance of cellular metabolic homeostasis (such as the ability to fine-tune cellular metabolism in response to stem cell maintenance and regeneration requirements) is central to normal growth, development, and senescence, as well as to the survival of organisms during periods of acute stress. These findings suggest that metabolic homeostasis is critical for maintaining the vital activities of stem cells and organisms and that dysregulation of metabolic homeostasis is closely associated with disease development. Stem cells can sense and respond to current metabolic demands via a variety of mechanisms to maintain metabolic homeostasis (Fig. 1). In this review, we summarized the factors affecting stem cell metabolic homeostasis, highlight the diversity of molecular regulatory mechanisms involved in the maintenance of stem cell metabolic homeostasis, discuss potential targets for aiding the metabolic regulation of stem cells, and provide insights into novel clinical therapies for diseases caused by imbalances in stem cell metabolic homeostasis.

## Concept of metabolic homeostasis in stem cells

Stem cells are primitive cells with self-replication, multidirectional differentiation, and homing capacities, which enables them to carry out various biological activities and thus play roles in normal physiological function.^4^ The basis of many vital functions is the transformation and transfer of matter and energy. Therefore, cellular energy metabolism is one of the most basic and important stem cell activities for maintaining intracellular homeostasis. In stem cells, energy is mainly derived from the synthesis and catabolism of energy-rich substances, such as glucose, fats, and amino acids. Glucose is also a key component that synthesizes many important substances in stem cells, such as nucleic acids and proteins, which are essential for the growth, division, and function of these cells. Lipids and amino acids can also act as signaling molecules that influence the signaling process of stem cells, thus affecting stem cell growth, differentiation, and apoptosis. In addition, amino acids provide a source of nitrogen for stem cell metabolism and life activities and participate in the composition of enzymes, hormones, and other active substances. One research group demonstrated that metabolites drive the differentiation of stem cells into organ-specific cells and that altering the metabolites in a cell can change the fate of that cell. Through single-cell metabolomics studies, a stem cell that had differentiated into an epidermal cell could be “reprogrammed” into a neural tissue cell and vice versa.^5^ Therefore, certain metabolic pathways, such as glycolysis, the tricarboxylic acid (TCA) cycle, and oxidative phosphorylation (OXPHOS), are core players in maintaining cellular metabolic homeostasis. Subsequently, stem cells can sense the changes in energy status through cellular energy sensors, such as adenosine monophosphate-activated protein kinase (AMPK) and mechanistic target of rapamycin (mTOR). They regulate their metabolic levels through a three-dimensional cellular metabolic-regulatory network, which exhibits traits such as interdependence, mutual constraints, and coordination so that they can remain in dynamic equilibrium under normal physiological conditions. This concept underlies the metabolic homeostasis of stem cells.

## Processes affecting metabolic homeostasis in stem cells

Within stem cells, complex interactions exist between pathways that maintain cellular metabolic homeostasis and energy regulation, which are necessary for cell survival. For example, the catabolism of glycogen produces glucose, which can enter the TCA cycle to generate energy, and certain intermediates in the TCA cycle can be converted into substances required for other metabolic pathways.^6^ Stem cells have the flexibility to switch between different metabolic processes to adapt to various conditions or stresses. Several instances of this phenomenon have been described in the literature. For example, the pluripotency of mesenchymal stem cells (MSCs) and hematopoietic stem cells (HSCs) is dependent on glycolysis, which switches to OXPHOS during cellular differentiation.^7^ During their differentiation into endodermal or mesodermal cells, the metabolic activity of pluripotent stem cells (PSCs) shifts to OXPHOS; however, during the initial stages of differentiation toward specific ectodermal fates, the rate of glycolysis remains high.^8^ Moreover, inhibition of mitochondrial glutamine metabolism will attenuate muscle stem cell proliferation and reactivate the transcription of self-renewal-related transcripts, thereby reducing stem cell heterogeneity and establishing a stem cell population with self-renewal functions.^9^ Increased expression of amino acid transporters mediates the uptake of leucine and glutamine, which is thought to stimulate mTOR complex 1 (mTORC1) activity and reduce reactive oxygen specie (ROS) in cells through the production of glutathione.^10^ In addition, metabolic intermediates can modulate epigenetic and gene expression via the regulation of chromatin-modifying enzymes, ultimately affecting cell fate.^11^ These interactions form a complex network that greatly influences stem cell metabolic homeostasis.

### Glucose metabolism

**Fig. 2:**
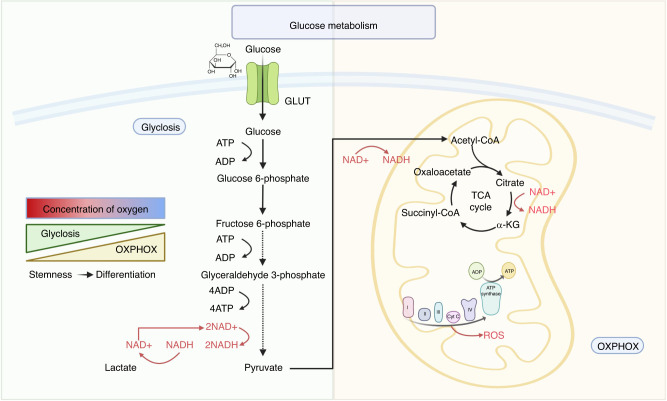
Glucose metabolism in the metabolic homeostasis of stem cells. Glycolysis and oxidative phosphorylation are the main sources of ATP in stem cells. Stem cells usually exist in anaerobic or hypoxic environments and therefore rely primarily on glycolysis for glucose metabolism. However, differentiated cells are often located in places where oxygen is more abundant, and the main mode of glucose metabolism shifts to oxidative phosphorylation. Created with BioRender. GLUT glucose transporter, ADP adenosine diphosphate, ATP adenosine triphosphate, NAD + nicotinamide adenine dinucleotide, NADH reduced nicotinamide adenine dinucleotide, ROS reactive oxygen species, α-KG α-ketoglutarate, OXPHOS oxidative phosphorylation

Based on findings from various studies on stem cells, glycolysis is now recognized as a central process in stem cell energy metabolism, with the mitochondria being key participants in this process. Stem cells mainly obtain adenosine triphosphate (ATP) through two metabolic pathways: glycolysis and OXPHOS. Intermediates from these pathways play important regulatory roles in the biological activities of stem cells. Therefore, altering the balance between these two pathways can affect stem cell function and their potential for generating differentiated progeny (Fig. 2).

#### Glycolysis

Glycolysis is a rapid process that converts a single glucose molecule into two pyruvate molecules to obtain a net gain of two ATP molecules. The pyruvate produced during glycolysis is further metabolized in two possible ways. Pyruvate can be converted to lactate and excreted from the cell. Alternatively, under well-oxygenated conditions, pyruvate can be transported to the mitochondria and oxidized to acetyl-coenzyme A to enter the TCA cycle and drive OXPHOS. Glucose itself is a key molecule that promotes specific pathways, such as the pentose phosphate pathway (PPP) and serine synthesis pathways. The PPP generates precursors for nucleotide and nonessential amino acid biosynthesis and strong reducing agents for anabolism and to counteract oxidative stress. The serine synthesis pathway contributes to the biosynthesis of nucleotides, amino acids, and lipids and maintains stem cell redox homeostasis.^12,13^ Thus, despite the high energy efficiency of OXPHOS, many stem cells have higher glycolytic capacity than differentiated cells, including bone marrow mesenchymal stem cells (BMSCs),^14,15^ embryonic stem cells (ESCs),^16^ the PSCs,^7,17^ and HSCs,^7,18–20^ to support cell growth and division. Inhibition of glycolysis in stem cells leads to cell cycle arrest and apoptosis but does not affect myofibroblast proliferation.^21^ This finding suggests that although glycolysis may be of some importance in all cells, it is particularly important for the maintenance of metabolic homeostasis in stem cells. Furthermore, metabolic regulation in the niche is important for stem cell function. Intestinal stem cells in proliferating embryonic-like spheres lack differentiated cell types and are primarily dependent on glycolysis, but those in miniature intestinal organs with defined crypt domains shift to depend on OXPHOS.^22^ Additionally, the specific metabolic pathways utilized by stem cells are influenced by their surrounding environment.^7,23^ Although most fully differentiated cells are distributed in tissues and organs with an adequate supply of oxygen, stem cells are believed to exist in ecological niches with low-oxygen tension, such as in the bone marrow and injured tissues. However, the preference of tumor stem cells for glycolysis is partially explained by defects in mitochondrial function, leading to an increased dependence on glycolysis, which is termed the Warburg effect.^24,25^

Increased glycolysis appears to promote stem cell activation in both the healthy and injured states. Knockdown of the mitochondrial pyruvate carrier (MPC) in hair follicle stem cells (HFSCs) and intestinal epithelial stem cells (IESCs) results in a marked bias for glycolysis alongside an increase in lactate production, which enhances stem cell proliferation.^26,27^ When factors such as aging and drug-related injuries lead to the loss of the long-term stemness of hematopoietic stem cells (HSCs), inhibition of sphingosine kinase 2 (Sphk2) effectively improves the cellular metabolic state of hypoxyglycolysis, which enhances the stemness maintenance and regenerative repair capacity of HSCs and effectively delays aging.^28^ Moreover, after novel coronavirus-related pneumonia and lung injury, alveolar stem cells showed significantly enhanced levels of 6-phosphofructo-2-kinase/fructose-2,6-biphosphatase 2 (PFKFB2), a key enzyme in glycolysis, indicating that glycolytic energy metabolism was increased. This increase may promote alveolar stem cell differentiation for alveolar regeneration and accelerate the regression of pulmonary fibrosis.^29^ In addition, skeletal muscle stem cells, also known as SCs, are activated when muscle injury occurs. This occurs because increased glycolysis under these conditions leads to increased histone acetylation by increasing the availability of acetyl-coenzyme A36 and decreasing the level of nicotinamide adenine dinucleotide (NAD+).^30^ This inhibits the activity of the NAD + -dependent histone deacetylase sirtuin 1 (SIRT1) and promotes the expression of muscle-generating genes, ultimately facilitating differentiation and inhibiting the regeneration of muscle fibers.^30^

Therefore, glycolysis may be a switch that determines the self-renewal and differentiation capacities of stem cells. However, current glycolysis-targeting drugs mainly focus on glucose transporter proteins, hexokinases, and pyruvate kinases, and the highly variable metabolic state of glycolysis in individual stem cells remains an obstacle to the design of therapeutic interventions.

#### Mitochondrial function and oxidative phosphorylation

Mitochondrial OXPHOS is a complex and important biochemical process and one of the energy conversion pathways necessary to maintain the vital activities of stem cells. The OXPHOS pathway is supported by five enzyme complexes that make up the electron transport chain (ETC): complex I, complex II, complex III, complex IV, and complex V. The chemo-permeable proton gradient generated by complexes I–IV is subsequently utilized by complex V, which catalyzes the phosphorylation of adenosine diphosphate (ADP) to produce ATP. Some electrons “leak out” of the electron transport chain, thereby ROS. Although stem cells may be less dependent on OXPHOS compared with differentiated cells, severe defects in mitochondrial function, the electron transport chain, or TCA cycling enzymes impair the self-renewal capacities of stem cells, particularly their ability to regenerate after tissue injury.^3^ Therefore, OXPHOS within the mitochondria is also a key component in maintaining metabolic homeostasis and the normal physiological functions of stem cells.

Increased mitochondrial OXPHOS capacity supports the metabolic needs of stem cells. Nestin+ BMSCs can increase energy production in leukemic stem cells (LSCs) via increased TCA cycling and OXPHOS, which provides LSCs with the key antioxidants needed to balance leukemogenesis and ROS levels during chemotherapy.^31^ This process is a potential target for adjuvant therapy in acute myeloid leukemia (AML). Deletion of the mitochondrial phosphatase protein tyrosine phosphatase mitochondrial 1 (PTPMT1), a phosphatase and tesin homolog (PTEN)-like phosphatase encoded by nuclear DNA and localized to the inner mitochondrial membrane via its N-terminal amino acids 1–37, in HSCs allows for its phosphatidylinositol substrate to directly potentiate fatty acid-induced activation of mitochondrial uncoupling protein 2 (UCP2), which alters the metabolic homeostasis of the mitochondria of HSCs, thus preventing their differentiation.^32^ In addition, PTPMT1 deficiency in irradiated mice disrupted the integrity of mitochondrial membranes and affected mitochondrial respiration in HSCs, which in turn impaired their ability to participate in hematopoietic repair.^32^ In addition, oxygen consumption and endogenous ATP production by MSCs (mesenchymal stem cells) are significantly increased during osteogenic differentiation, and inhibition of mitochondrial function hinders osteogenic differentiation. Therefore, pharmacological interventions targeting mitochondrial OXPHOS pathways have emerged as potential therapeutics for osteoporosis.^33,34^ Meanwhile, with recent advances in biomaterials research, the use of iron oxide nanoparticles to increase the mitochondrial “capacity” and “output efficiency” of MSCs has been found to achieve efficient and sustained mitochondrial “recharging” by targeting damaged lung cells, which has been shown to be effective in a mouse model of progressive fibrosis.^35^

In recent years, studies of mitochondrial OXPHOS have not exclusively focused on its role in energy production. Metabolites of the TCA cycle, such as acetyl-coenzyme A, α-ketoglutarate, S-adenosylmethionine, and nicotinamide adenine dinucleotide, have emerged as key regulators required for the post-translational modifications of DNA and histones.^36–39^ In stem cells, the reduced nicotinamide adenine dinucleotide (NAD+/NADH) ratio regulates the activity of a variety of enzymes (such as histone deacetylases) and influences gene expression. NAD+ levels in stem cells decline with age, and dietary supplementation with NAD+ precursors improve the function of senescent stem cells in the hematopoietic system, brain, muscle, and hair follicles.^3,40,41^ Several studies have also identified NAD+ metabolism as the basis of immunomodulatory functions in MSCs and found that nicotinamide-phosphate ribosyltransferase (NAMPT)-mediated NAD+ metabolism activates and maintains the aerobic glycolytic program in the cytoplasm through driving the succinate-hypoxia-inducible factor 1-alpha (HIF1α) axis in the mitochondria, which in turn shapes the immunosuppressive function of MSCs and exerts an important therapeutic impact on disease.^42^ In ESCs, mitochondria are in a “hyperactive” state, characterized by efficient ATP production; OXPHOS can couple the amino-hexose synthesis pathway to regulate the self-renewal and multidirectional differentiation potential of PSCs via aminoacyl glycosylation by pluripotency factors.^43^ In addition, the nuclear localization of pyruvate dehydrogenase E1 subunit alpha 1 (PDHA1), a TCA cycle enzyme in PSCs, promotes the direct synthesis of acetyl-coenzyme A in the nucleus, which provides a reaction substrate for histone acetylation. This thereby increases the occurrence of acetylated lysine 9 on histone H3 (H3K9ac) and acetylated lysine 27 on histone H3 (H3K27ac) modifications in the transcriptional start sites and enhancer regions of pluripotency-related genes, which are critical for pluripotency. These findings collectively indicate the physiological significance of OXPHOS in early development.^44^

Taken together, mitochondrial OXPHOS has profound roles in implications stem cell metabolic homeostasis and preventing diseases associated with dysregulated stem cell metabolic homeostasis. As such, mitochondrial OXPHOS has great practical value as a target in stem cell therapy. However, our understanding of the relevant signals from mitochondrial OXPHOS that influence stem cell function is currently limited. Future studies should also aim to address how mitochondrial OXPHOS is spatially and temporally regulated in stem cells. Finally, whether stem cells from different tissues and organs communicate with each other regarding mitochondrial metabolism and work together to coordinate the overall metabolic and homeostatic processes of the organism remains unclear.

### Lipid metabolism

**Fig. 3:**
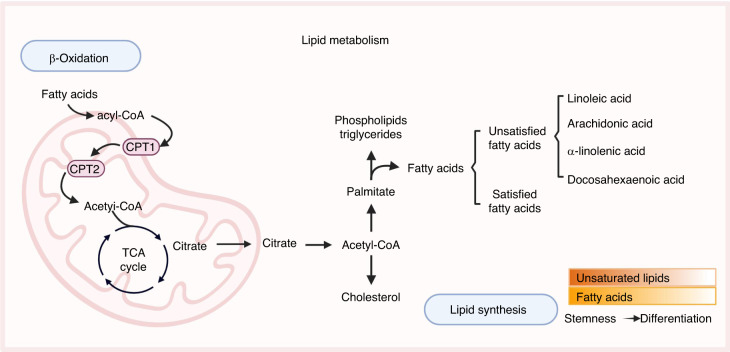
Lipid metabolism in the metabolic homeostasis of stem cells. Lipids play crucial roles in stem cell biology, including structural support, energy storage, and signaling. FAO occurs mainly in mitochondria and is an important source of energy. During FAO, fatty acids are converted to acetyl-CoA, which enters the TCA cycle to produce ATP. Lipid synthesis process primarily occurs in the endoplasmic reticulum and the cytoplasm, and involves the condensation of acetyl-CoA and malonyl-CoA monomers to form fatty acids, which are then incorporated into a variety of lipid molecules. Stem cells exhibit elevated levels of unsaturated lipids and fatty acids in their lipid metabolism, and these levels decline upon differentiation. Created with BioRender. CPT carnitine palmitoyltransferase, TCA tricarboxylic acid cycle

Lipids include fatty acids, triglycerides, cholesterol, and phospholipids. Fatty acids are classified into saturated and unsaturated fatty acids, which can be oxidized and decomposed in the mitochondria to release energy for normal life activities. Lipid metabolism is another important metabolic pathway in stem cells that plays key roles in cell membrane structure, energy metabolism, and signal transduction (Fig. 3).

Fatty acids are essential for the pluripotency, proliferation, and survival of stem cells. In contrast to differentiated cells, the lipid profile of ESCs is characterized by elevated levels of unsaturated lipids and fatty acids; inhibition of the eicosanoid signaling pathway promotes pluripotency and maintain the level of unsaturated fatty acids.^45,46^ Unsaturated fatty acids, such as linoleic acid, arachidonic acid, α-linolenic acid, and docosahexaenoic acid (DHA), activate the Wnt, C-X-C chemokine receptor type 4 (CXCR4), and Notch pathways in the bone marrow microenvironment.^47^ By regulating the activity of intracellular metabolic enzymes, these pathways promote lipid metabolism and enhance the colony formation and proliferative capacities of HSCs.^48^ In addition, during fracture healing, fatty acids in the blood act as signaling molecules to induce the differentiation of skeletal stem cells (SSCs) into osteoblasts.^49^ If there is a paucity of fatty acids and/or nearby blood vessels, forkhead box O (FOXO) signaling is activated; nuclear localization of FOXO promotes the expression of SY-box transcription factor 9 (SOX9), which not only results in the eventual formation of cartilaginous tissue by the stem cells but also inhibits fatty acid oxidation (FAO) to regulate cellular metabolic homeostasis.^49^ These alterations help the cells adapt to the new avascular environment. Omega-3 fatty acids induce adipose stem cells (ASCs) division when bound to free fatty acid receptor 4 (FFAR4) on the surface of the primary cilia of ASCs; this promotes the production of additional adipocytes for energy storage, which is healthier than storing excess fat in existing adipocytes and allows for the maintenance of fat homeostasis.^50^ Moreover, special enzymes, such as fatty acid synthase (FASN), are primarily responsible for the formation of fatty acids, and mutations in FASN can lead to the accumulation of fat within cells, which puts neural stem cells (NSCs) under stress; this stress can reduce the ability of NSCs to divide.^51^ This further revealed the mechanism by which lipid metabolism regulates NSC activity and affects brain development.

With the increasing application of single-cell transcriptome technology in recent years, genes related to FAO metabolic-regulatory pathways, including carnitine palmitoyltransferase 1a (CPT1a), promyelocytic leukemia (PML), and peroxisome proliferator-activated receptor delta (PPARD), have been demonstrated to be highly expressed in stem cells, which suggests that FAO is essential for the maintenance of metabolic homeostasis in various types of stem cells.^52^ CPT1a knockdown results in the inhibition of FAO and reduced acetyl-coenzyme A synthesis, which inhibits the histone acetylation of differentiation-related factors in HSCs; collectively, these changes lead to both an increase in the proliferative capacity and a decrease in the differentiation capacity of HSCs.^53^ Moreover, the expression of fatty acid metabolism-related pathway genes are significantly altered during stereotyped endodermal differentiation. Inhibition of FAO inhibits the differentiation of ESCs into the stereotyped endoderm, whereas inhibition of fatty acid synthesis promotes the differentiation of ESCs into the stereotyped endoderm.^54^ These results reveal the important role of cellular lipid metabolism homeostasis in the genealogical specialization of human ESCs. In addition, some researchers have observed that fasting in the intestine promotes the proliferation of intestinal stem cells (ISCs) by regulating FAO, thereby providing opportunities for targeted therapies to improve aging-related tissue homeostasis.^55^

A high-fat diet (HFD) leads to the strong activation of peroxisome proliferator-activated receptor gamma (PPARγ)-2, a key regulator of adipogenesis and adipose tissue development, which impairs osteoblastogenesis while enhancing bone marrow adipogenesis. Antibiotic treatment partially mitigated the effects of high-fat diet on the bone marrow niche, and fecal transplantation of high-fat diet mice transferred this effect to normal mice.^56,57^ This finding suggests that HFD affects the bone marrow niche through altering gut microbiota and osteoblast–adipocyte homeostasis. Thymoquinone (TQ), a natural quinone (C_10_H_12_O_2_), has been reported to possess anti-inflammatory, antioxidant, antidiabetic, and antihyperlipidemic properties and to reduce the differentiation of adipose-derived stem cells (ADSCs) to adipocytes through inhibiting the expression of PPARγ and FASN.^58^ By regulating PPAR-γ, a key target of lipid metabolism, TQ may be a potential anti-obesity compound. In conclusion, lipid metabolism, which serves as a primary pathway for cellular energy storage and production, is involved in the maintenaining the metabolism and functional homeostasis of stem cells; however, due to the diversity and complexity of lipid metabolism pathways, their intrinsic regulatory mechanisms require further exploration. Targeting key regulators of fatty acid metabolism may improve the regenerative ability of stem cells and promote the recovery of stem cell function after disease.

### Amino acid metabolism

**Fig. 4:**
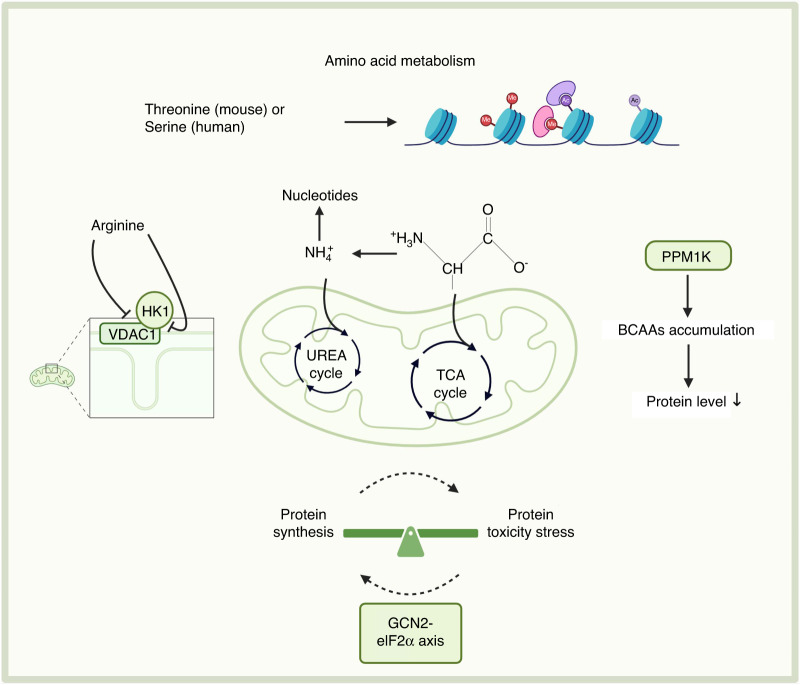
Amino acid metabolism in the metabolic homeostasis of stem cells. Amino acid metabolism involves the synthesis and degradation of amino acids and plays a crucial role in various processes in stem cells. The GCN2-eIF2α axis, PPM1K, arginine, and serine play important roles in stem cell homeostasis through amino acid metabolism. Created with BioRender. PPM1K protein phosphatase Mg^2 +^ /Mn^2+^-dependent 1K, GCN2-eIF2α axis general control nonderepressible 2-eukaryotic initiation factor 2α axis, HK1 hexokinase 1, VDAC1 voltage-dependent anion channel 1

Amino acids are the basic units of proteins and are important metabolic substances within the cell. Amino acid metabolism refers to the uptake, conversion, and utilization of amino acids within stem cells. Stem cells can metabolize amino acids to synthesize proteins and produce energy, which aids in maintaining the homeostasis of the intracellular environment (Fig. 4).

In recent years, deletion of general control nonderepressible 2 (GCN2), a classical sensor of amino acid deficiencies, has been found to lead to activation of the Scr-AKT signaling pathway, which in turn results in the over-enhancement of HSCs mitochondrial energy metabolism and impairs the long-term hematopoietic capacity of HSCs.^59^ This study has elucidated the association and molecular mechanism between energy metabolism and maintenance of protein homeostasis in HSCs and revealed that the metabolite nicotinamide ribose can promote protein homeostasis in HSCs via regulating amino acid catabolism to protect long-term hematopoiesis. Therefore, controlling protein synthesis and proteotoxic stress in homeostatic HSCs by targeting the GCN2-eukaryotic initiation factor 2α (eIF2a) axis could be a novel strategy for expanding HSCs and maintaining their long-term function.^59^ This strategy would be clinically useful for promoting HSC expansion in vitro and enhancing the hematopoietic reconstitution of HSCs after transplantation. Moreover, knockdown of protein phosphatase Mg^2 +^ /Mn^2 + ^-dependent 1 K (PPM1K), the rate-limiting enzyme for the degradation of branched-chain amino acids (BCAAs), leads to massive accumulation of cytoplasmic BCAAs.^60^ PPM1K knockdown also significantly enhances E3 ubiquitin ligase CDC20-mediated downregulation of various proteins, thus causing impaired and dysfunctional metabolism and HSC quiescence.^60^ These mechanisms may play an important regulatory role in malignant transformation to leukemia. Similarly, excessive accumulation of BCAAs is detrimental to MSC retention and is an important alteration in the heart post-ischemia.^61^ Therefore, enhancing the catabolic capacity of BCAAs in MSCs using appropriate methods may be a feasible strategy for improving the adaptation of bone marrow MSCs to an ischemic myocardial environment, which may optimize the cardiac repair effects of bone marrow MSC-based therapies. In addition, extracellular glutamine depletion, glutaminase inhibition (conversion of glutamine to glutamate via ammonia release), and glutamyl transaminase inhibition (linking glutamine catabolism to the TCA cycle and amino acid synthesis) strongly reduced the colony formation and proliferative capacities of BMSCs.^62^ BMSCs were characterized by the expression of CD73, an extracellular 5-nucleotidase that catalyzes the final step in the conversion of extracellular ATP to adenosine. Extracellular ATP has been suggested to be a danger signal that promotes proliferation and migration and affects the differentiation of HSCs. Also, BMSCs secrete large amounts of valine, which is essential for the maintenance and proliferation of HSCs. However, the relative importance of niche-derived versus direct dietary supply of valine for HSCs remains to be determined.^14^ Another research group observed that glutamine metabolism is also an important source of energy during the differentiation of CD34^+^ HFSCs to CD34^-^ precursor cells, although the reverse process (differentiation of precursor cells to HFSCs) requires reduced glutamine metabolism.^63^ Therefore, inhibition of mitochondrial glutamine metabolism by suppressing glutaminase expression could play an important regulatory role in the homeostasis of HFSCs during the hair regeneration cycle. As for NSCs, it has been revealed that arginase-II knockdown was revealed to lead to elevated intracellular arginine concentrations, inhibits interactions between hexokinase 1 (HK1) and voltage-dependent anion-selective channel protein 1 (VDAC1), and affects the attachment of HK1 to the mitochondria, which results in the overactivation of adult NSCs and neurological disorders.^64^ Recently, the critical importance of threonine (mouse) or serine (human) metabolism for the growth and differentiation of ESCs and induced PSCs was identified, as these amino acids are precursor molecules for histone methylation and acetylation.^65^

In summary, current research on amino acid metabolism in stem cells mainly focuses on specific types of stem cells, such as HSCs, MSCs, and HFSCs. Research on other types of stem cells is not as in-depth, necessitating the exploration of the metabolic sensitivity of different stem cells to amino acids. In the future, stem cell-specific amino acid metabolomics analyses will lead to a deeper understanding of the regulatory mechanism of amino acid metabolic homeostasis. Future studies in these directions will generate novel ideas and methods to promote the differentiation and regeneration of stem cells.

## Molecular mechanisms of metabolic homeostatic regulation in stem cells

**Fig. 5:**
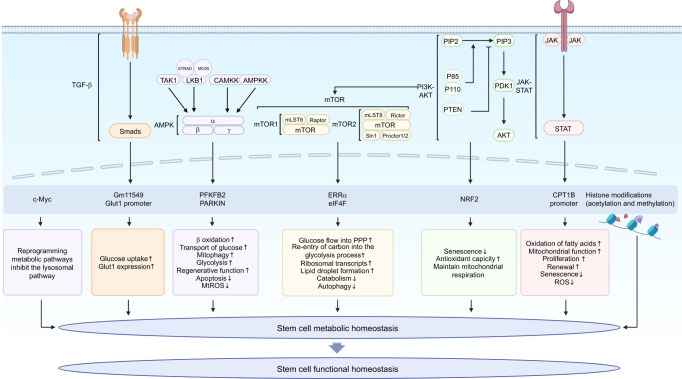
Molecular mechanisms of metabolic homeostatic regulation in stem cells. Stem cells regulate metabolic levels through a three-dimensional cellular metabolic-regulatory network that regulates downstream target genes and transcription factors to carry out a variety of metabolic processes, including glucose uptake, β-oxidation, autophagy, and so on, so as to maintain a dynamic balance under normal physiological conditions. Created with BioRender. TGF-β transforming growth factor-β, AMPK adenosine monophosphate-activated protein kinase, LKB1 liver kinase B1, STRAD sterile 20 related adaptor protein, MO25 mouse protein 25, CAMKK2 calcium-sensitive kinase 2, TAK1 transforming growth factor β-activated kinase-1, AMPKK AMP-activated protein kinase kinase, mTOR mammalian target of rapamycin, mTORC1 mTOR complex 1, mTORC2 mTOR complex 2, mLST8 mTOR-associated protein LST8 Homolog, Sin1 stress-activated protein kinase-interacting protein, PI3K-AKT phosphoinositide-3-kinase AKT, PIP2 phosphatidylinositol-4,5-bisphosphate, PIP3 phosphatidylinositol-3,4,5-trisphosphate, PTEN phosphatase and tensin homolog deleted on chromosome ten, PDK1 phosphoinositide-dependent kinase-1, STAT signal transducer and activator of transcription, PFKFB2 6-phosphofructo-2-kinase/fructose-2,6-biphosphatase 2, ERRα estrogen-related receptor α, eIF4F eukaryotic initiation factor 4F, NRF2 nuclear factor erythroid 2-related factor 2, PPP pentose phosphate pathway, mtROS mitochondrial ROS

Stem cell metabolism and energy regulation are important processes in living organisms and are realized through various molecular mechanisms. Through the regulation of these molecular mechanisms, stem cells maintain a normal energy state and can sustain their normal biological functions, thereby enabling human health. Therefore, studying the specific molecular mechanisms that regulate metabolic homeostasis in stem cells is important (Fig. 5).

### Adenosine monophosphate-activated protein kinase

Adenosine monophosphate-activated protein kinase (AMPK), an important kinase in the regulation of energy homeostasis, is a central regulator of cellular and organismal metabolism in eukaryotic organisms and can be activated by a variety of stimuli, including cellular stress, motility, and many hormones and substances, which affect cellular metabolic processes.^66^ Studies have shown that AMPK is responsible for regulating the input and output capacities of stem cells and maintaining their physiological functions.^67,68^

In mammals, although a complete structural map of the AMPK heterotrimer is not yet available, the structures of the various AMPK domains are well-defined. These include the α (α1, α2) subunits, which play a catalytic role, and the β (β1, β2) and γ (γ1, γ2, γ3) subunits, which play a role in maintaining the stability of the trimer and specifically act on the substrate.^69–72^ The N-terminal end of the α subunit includes a conserved serine/threonine kinase region, which contains a conserved threonine (Thr-172) site.^73^ Phosphorylation of this conserved threonine site is required for kinase activity. Four upstream kinases of AMPK have been identified; these include tumor suppressor liver kinase B1 (LKB1), AMP-activated protein kinase kinase (AMPKK), transforming growth factor kinase-1 (TAK1), and Ca2 + -dependent calcium/calmodulin-dependent protein kinase 2 (CaMKK2).^74^ After the upstream kinase CAMKK2 is activated by intracellular calcium ions and LKB1 forms a heterotrimer with sterile 20 related adaptor protein (STRAD) and mouse protein 25 (MO25), the AMPK machinery is activated. In turn, this machinery regulates a wide range of processes, including glucose metabolism, lipid metabolism, protein metabolism, autophagy, and mitochondrial homeostasis.^75,76^ Therefore, as a cellular energy receptor, when ATP levels decrease in stem cells, activation of AMPK inhibits the mTOR pathway to negatively regulate ATP-consuming biosynthetic processes by inhibiting gluconeogenesis, lipid, and protein synthesis. Simultaneously, it positively regulates signaling pathways that increase cellular ATP production by promoting FAO and glucose transport to restore a more favorable energy balance state.

Pyruvate, which is produced during glycolysis as mentioned earlier, is a key molecule in stem cell metabolism and is widely used for the culture of ESCs.^77^ Elevated exogenous pyruvate levels alter the metabolic profile of ESCs and enhance oxidative metabolism via modulation of the AMPK pathway, which in turn promotes mesoderm- and endoderm-lineage-specific differentiation.^78^ In addition to glucose metabolism, fatty acid metabolism plays a key role in the final endodermal differentiation of human ESCs.^54^ Enhanced fatty acid β-oxidation and reduced adipogenesis were observed as a result of phosphorylation of acetyl-CoA carboxylase, an adipogenic enzyme, by AMPK.^54^ Thus, inhibition of fatty acid synthesis by AMPK agonists is instrumental in promoting and regulating human endodermal differentiation. Alveolar epithelial type II cells (AT2), which dominate alveolar regeneration as alveolar stem cells, differentiate into alveolar epithelial type I cells (AT1), which perform gas exchange. Some studies have activated the AMPK-PFKFB2 signaling pathway using small-molecule compounds or genetic methods for sufficient energy production, which has been confirmed to be effective in promoting the regenerative function of senescent stem cells.^29^ These findings provide an important direction for the development of therapeutic approaches for senescence-associated lung diseases. Within bone, stabilizing mutations in Notch2 can lead to Hajdu–Cheney syndrome, which is characterized by early onset osteoporosis^79^; however, the underlying mechanism remains incompletely understood. Recent studies have revealed that Notch inhibits the expression of glycolytic and mitochondrial complex I genes, leading to significant reductions in mitochondrial respiration, superoxide production, and AMPK activity.^80^ In contrast, reactivation of AMPK antagonizes the glycolytic processes inhibited by Notch, thereby promoting the osteogenic differentiation of BMSCs and ultimately alleviating osteoporosis.^80^ In addition to diseases where genetic mutations are present, the exact pathways through which alcohol interferes with homeostasis within the femoral head remain to be elucidated. Previous studies have suggested that alcohol affects the balance between cholesterol and lipid metabolism in BMSCs by modulating the AMPK pathway, which leads to pathological changes characterized by osteoclast death and sparse bone microstructure.^81^ Furthermore, AMPK serves as a crucial target of metformin, and recent studies have shown that the AMPK pathway mediates the effects of metformin on the metabolism of stem cells in the stomach. Metformin administration promotes their differentiation into acid-secreting gastric lining cells and further induces maturation. These findings provide important insights into why metformin increases acid secretion and reduces the risk of developing gastric cancer.^82^ In the field of stem cell therapies, umbilical cord-derived MSCs (UC-MSCs) can induce mitochondrial autophagy in hepatocytes by directly activating the AMPK pathway, leading to phosphorylation of PTEN-induced putative kinase-1 (PINK1) and upregulation of Parkin levels. These alterations are critical for scavenging mitochondrial reactive oxygen species (mtROS), decreasing mitochondrial fragmentation, and inhibiting apoptosis; thus, targeting this pathway has potential in the treatment of hepatic ischemia-reperfusion injury.^83^

Recently, AMPK substrates have attracted research attention. Although AMPK and its related family members typically phosphorylate a common set of substrates in stem cells, the conditions under which AMPK or its related family members are active vary. Defining the activity of each AMPK-associated kinase in different stem cell types and conditions remains a key goal in resolving the role of AMPK substrates in growth and metabolic homeostasis. Subsequently, as the list of AMPK substrates has been refined, it has become increasingly clear that AMPK signaling acts in concert with multiple signaling pathways.^84^ The convergence of these pathways suggests that a small set of rate-limiting regulators can act as major coordinators of cell growth, metabolism, and ultimately cell fate. In the future, studies should primarily aim to define targets that are necessary and relevant for the beneficial effects of AMPK activation in stem cells under pathological conditions. Identifying these key targets will allow us to exploit endogenous mechanisms to restore metabolic homeostasis in stem cells.

### Phosphoinsoitide 3-kinase-AKT

As a major regulatory molecule of stem cell metabolism and growth, phosphoinositide-3-kinase (PI3K) controls important biological processes, such as stem cell nutrient uptake, energy production, cofactor production, and macromolecule biosynthesis.^85^ PI3K can be regarded as a central force in driving stem cell metabolism. The PI3K-AKT pathway is mediated by the phosphorylation of a serine or threonine in a series of downstream substrates. PI3K phosphorylates the key metabolite phosphatidylinositol-4,5-bisphosphate (PIP2) to produce phosphatidylinositol-3,4,5-bisphosphate (PIP3), and PIP3 subsequently recruits downstream signaling proteins, including the serine/threonine kinase Akt, lipid phosphatase PTEN, and phosphatidylinositol-3-dependent protein kinase-1 (PDK1).^86^

In recent years, multiple studies have identified the PI3K/AKT pathway as a key target metabolic signaling pathway for improving stem cell survival. Massive cell death in harsh environments is an obstacle to the successful transplantation of MSCs. One study demonstrated that platelet-rich clot release (PRCR) preconditioning could increase paracrine factor levels through the PI3K-AKT signaling pathway, thereby reprogramming MSCs to tolerate unfavorable microenvironments and enhancing their regenerative capacities; this method can be used to improve the therapeutic outcomes of clinical MSC transplantation.^87^ Moreover, a report showed that hepatocyte growth factor (HGF) and stem cell factor (SCF), which are key factors in maintaining the properties of BMSCs, can play roles in maintaining mitochondrial metabolic function through the expression of proteins associated with the PI3K-AKT signaling pathway. These findings provide new evidence for optimizing potential modalities to optimize the long-term culture of MSCs.^88^ Rg1, a natural ginseng extract, activates nuclear factor erythroid 2-related factor 2 (NRF2) by interacting with the PI3K-AKT pathway; administration of Rg1 can prevent the aging of MSCs and improve their antioxidant capacity.^89^ As such, Rg1 is expected to serve as a promising regenerative drug to assist in the treatment of tissue aging.^89^ Insulin is an important growth factor for the survival and self-renewal of ESCs^90^; however, its role in ESC energy metabolism remains unclear. Sustained insulin signaling synergizes pyruvate and glutamine to maintain mitochondrial respiration via activation of PI3K-AKT signaling, which promotes cell survival.^91^ Meanwhile, in the field of of HSC research, the limited in vitro expansion of HSCs remains a major obstacle to the widespread therapeutic application of HSC transplantation. It has recently been revealed that combinations of a PI3K-AKT activator with a thrombopoietin receptor agonist and the pyrimidine-indole derivative UM171 are sufficient to stimulate the expansion of umbilical cord blood HSCs, enabling successful colonization and growth in xenotransplantation trials. These findings have helped advance multiple potentially life-saving HSC transplantation-related therapies, which are currently in clinical development.^92^

In addition to influencing stem cell survival, the PI3K-AKT pathway is one of the metabolic mechanisms that regulates stem cell quiescence. Strict regulation of HSC homeostasis ensures lifelong hematopoiesis and prevents the development of blood cancers. Inositol-triphosphate-3-kinase B (ITPKB) was found to promote HSC quiescence and function, inhibit HSC activation by limiting cytokine-induced PI3K-AKT signaling, and maintain homeostasis in hematopoietic progenitor cells.^93^ Three-dimensional (3D) cultures can reflect more biological properties of stem cells in vitro. The slow rate of stress relaxation in hydrogels puts MSCs in a reversible quiescent state owing to attenuated PI3K-Akt activation; this state allows MSCs to process substrates under decreased deformation to maintain their stemness.^94^

In conclusion, the PI3K-AKT signaling pathway plays a critical role in cellular metabolic reprogramming, and its activation and inhibition are closely related to the functional homeostasis of a variety of stem cells. Future studies should aim to refine the molecular map of key regulatory nodes that connect the PI3K-AKT signaling network and other metabolic networks, which will help reveal metabolic dependencies and guide the development of novel stem cell therapeutic strategies. With the discovery of novel metabolites, metabolic enzymes have provided many novel targets against which specific molecular targets can be developed. Therefore, we expect to see the emergence of personalized therapeutic regimens targeting the PI3K-AKT signaling pathway in the near future, which will provide better treatment options for patients.

### Mammalian target of rapamycin

The mammalian target of rapamycin (mTOR) is a highly conserved serine/threonine protein kinase that exists downstream of PI3K/AKT in mammals. mTOR kinases are mainly divided into two distinct complexes: mTORC1 and mTOR complex 2 (mTORC2). These complexes have distinct structures that are regulated by different nutrient and environmental factors and can phosphorylate different substrates, thus playing distinct roles in the regulation of cellular metabolism and proliferation.^95^ As a regulator of responses to environmental and hormonal stress, mTORC1 activates anabolic processes (such as the synthesis of proteins, lipids, and nucleotides) when nutrients (including amino acids, glucose, cholesterol, and nucleotides) are abundant.^96^ Simultaneously, it inhibits the catabolic processes and autophagy of stem cells, all of which contribute to cell growth and proliferation. In contrast, under nutrient-deficient or stressful conditions, mTORC1 is inhibited, which in turn inhibits energy-consuming anabolic processes and promotes catabolic processes, thereby providing the nutrients and energy required for stem cell survival.^97^ mTORC2 mainly acts as an effector of PI3K signaling and is involved in the regulation of cell survival and cytoskeleton formation via its enhanced activation in response to insulin, insulin-like growth factor 1 (IGF-1), and leptin.^98^ Therefore, mTOR, a central regulator of metabolism, integrates multiple nutritional and hormonal signals to modulate metabolic homeostasis in stem cells.

mTOR signaling has been identified as a metabolic pathway that influences the differentiation of various stem cells. In the first step of the differentiation of MSCs into osteoblasts, the mitochondrial glutamine backfilling mechanism is necessary for glutaminase (GLS) to catalyze the production of glutamate from glutamine (Gln), and estrogen-related receptor alpha (ERRα) is a key gene involved in regulating mitochondrial function. Recently, it was revealed that the nutrient-sensing factor mTOR regulates GLS through the nuclear receptor ERRα and affects mitochondrial glutamine metabolism, thus providing the energy required for the synthesis of nucleic acids, proteins, and other biomolecules of MSCs. This process also enhances the osteoblastic differentiation of senescent MSCs and provides an important direction for future studies on how stem cell differentiation is induced and modulated.^99^ In addition, the microRNA miR-199-3p, which is found in exosomes isolated from adipose-derived MSCs, promotes the autophagy of chondrocytes by inhibiting the mTOR signaling pathway; this, in turn, upregulates cartilage matrix-synthesizing proteins and downregulates cartilage matrix-degrading proteins to promote the metabolic homeostasis of chondrocytes, effectively alleviating the degeneration of articular cartilage in osteoarthritis (OA) rats.^100^ Heterotopic ossification is usually caused by the emergence of osteoblasts in soft tissues and the eventual formation of bony tissues; however, the specific underlying mechanism is not well understood; nesfatin-1, an adipokine composed of 82 amino acids, can inhibit autophagy through the mTOR pathway and promote osteogenic differentiation and heterotopic ossification of rat tendon stem cells (TDSCs). Thus, nesfatin-1 may be a potential therapeutic target for the treatment of tendinopathy.^101^

Autophagy is a fundamental mechanism through which mTOR regulates metabolic homeostasis. However, it is unclear why the downregulation of autophagy leads to a significant reduction in the HSC pool. Research has shown that mTORC1 activation leads to increased cell size, glucose uptake, protein synthesis, and translation in autophagy-deficient HSCs, which is detrimental to the cells.^102^ Therefore, autophagy can maintain metabolic homeostasis in stem cells by regulating amino acid inputs and inhibiting the activity of the mTORC1 signaling pathway. These findings provide a theoretical basis for future therapies targeting this pathway. Moreover, several other studies on HSCs have found that mTORC1 activity is elevated in HSCs from aged mice, which is similar to the functional deficits observed in a mouse model of overactive mTORC1.^103,104^ Treatment of aged mice with rapamycin restored the functional homeostasis of HSCs and enhanced the immune responses to influenza infection.^105^ In another study, based on high-throughput sequencing of single-cell transcriptomes and two conditional Rictor (a key mTORC2 constituent) knockout mouse models specific for vascular endothelial and hematopoietic cells, it was shown that the mTORC2 signaling pathway plays a critical role in the formation and specialization of HSCs; however, it is not required for the maintenance of HSCs thereafter.^105,106^ This suggests that the metabolic processes associated with the mTOR signaling pathway play an important role in the cell-type transition from vascular endothelial cell specialization to the formation of HSC precursors.

In other stem cell systems, research has shown that HFSCs prefer glycolytic metabolism to glutamine metabolism for energy; this metabolic switch is triggered by a low-oxygen environment and Rictor signaling.^63^ The number of HFSCs is reduced in Rictor-deficient mice; however, the use of a glutaminase inhibitor restores HFSC function, implying that modifying the metabolic pathway may be an effective method for enhancing tissue regeneration.^63^ Other researchers have observed that mTOR-mediated regulation of ESC self-renewal comes mainly from the action of mTORC1 and that the specific mechanism is not related to genome-wide transcription; instead, regulation occurs through the protein complex eukaryotic translation initiation factor 4F (eIF4F), which synergistically regulates the translation of proteins in the cytoplasm and mitochondria.^107^ Thus, endogenous mTORC1 maintained self-renewal in ESCs without affecting pluripotency. Because adult stem cells and functional cell precursors are currently unable to achieve long-term stable expansion in vitro as ESCs, the activation of self-renewal in these cells while maintaining their differentiation potential will be of great significance in overcoming disease and aging.

Over the past few years, we have made great strides in our understanding of the physiological and metabolic processes controlled by mTOR signaling and the regulation of mTOR signaling by nutrients, metabolites, and hormones. However, many questions remain regarding the regulation of mTOR and the physiological role of mTOR signaling. For example, it remains unclear how many essential amino acids, glucose, and fatty acids act as signals for lysosomal mTORC1; in addition, little is known about the regulation of mTORC1 activity at non-lysosomal sites, and the complete signaling network downstream of the mTOR complex remains to be elucidated. In the future, as our ability to precisely regulate mTOR activity and its downstream processes increases, we expect to see various novel pharmacological approaches for specifically modulating mTOR with fewer side effects to promote human health.

### Janus kinase-signal transducer and activator of transcription

The anus kinase (JAK)-signal transducer and activator of transcription (STAT) pathway comprises three components: transmembrane receptors, receptor-associated cytoplasmic JAKs, and STATs.^108^ Different cytokines or glycoproteins have their own corresponding transmembrane receptors. A common feature of these receptors is that the receptor itself does not have kinase activity, but the intracellular segment has a binding site for JAKs; these binding sites enable extracellular to intracellular transmission of the corresponding signals.^109^ Activated JAK phosphorylates a conserved tyrosine residue of one STAT monomer, which then binds to the Src homology 2 (SH2) structural domain of another monomer, driving a conformational change that allows the phosphorylated STAT to translocate to the nucleus. This translocation directly initiates the transcription of metabolism-associated target genes, thereby affecting cellular metabolic homeostasis.^110^

HFSCs are long-lived cells in the hair follicle, and abnormal activation of HFSCs is a major cause of hair loss.^111^ The drug RCGD423 was found to activate the JAK-STAT signaling pathway in HFSCs to promote the expression of enzymes related to lactate metabolism, leading to an increase in the intracellular production of lactate, which promotes the activation of HFSCs and hair growth. Furthermore, although the JAK-STAT3 pathway may maintain the phenotype and function of tumor stem cells by regulating anabolic and catabolic metabolism, the exact pathway by which this is achieved remains unclear.^26^ Recently, it has been shown that human adipocyte-derived leptin, via activation of JAK-STAT3 signaling, promotes STAT3 phosphorylation and entry of p-STAT3 into the nucleus to bind to the carnitine palmitoyltransferase 1 B (CPT1B) promoter. This promotes its transcription and FAO, which in turn promotes the proliferation of breast cancer stem cells (BCSCs) and cancer cell drug resistance.^112^ Thus, inhibiting JAK, FAO, or leptin inhibits BCSC self-renewal and re-sensitizes cancer cells to chemotherapeutic agents. These findings have helped clarify the pathways that contribute to breast cancer growth and chemoresistance. Additionally, STAT3 regulates transcription in the nucleus and electron transfer and OXPHOS in the mitochondria. Studies have confirmed that STAT3 deficiency in HSCs leads to mitochondrial dysfunction, overproduction of ROS, and premature senescence of blood cells; hence, the mitochondrial function of STAT3 may be required to prevent stem cell senescence.^113^ Moreover, several downstream target proteins of the JAK-STAT signaling pathway have been identified to be involved in regulating the self-maintenance of male germline stem cells.^114^ However, how the JAK-STAT signaling pathway maintains the stem cell properties of germline stem cells remains to be explored. Recently, p115 was identified as a downstream target protein of the JAK-STAT signaling pathway, forming a positive feedback loop to maintain germline stem cells. These findings provide a new perspective for understanding how the JAK-STAT signaling pathway efficiently controls the regulatory mechanisms of stem cell maintenance.^115^ In addition, in the field of stem cell therapy, some studies have demonstrated that MSCs can control sepsis-induced inflammatory responses by regulating T-helper cells and inflammatory factors, thereby reducing tissue damage and protecting organ function.^116^ This ultimately improved the survival of rat models of aging and sepsis, in which inhibition of the JAK-STAT signaling pathway may be an important mechanism of action.^116^ Several metabolites, including fructose 1,6-bisphosphate (FBP), phosphoenolpyruvate (PEP), and sodium oxalate (OXA), can induce cellular glycolytic metabolism via STAT signaling, thus stimulating MSC proliferation. This is an effective means of enhancing the homeostatic maintenance of MSCs and is of great significance for the study of diseases that may benefit from stem cell therapy.^117^

At present, structural information about cytokine receptors and STAT complexes remains sparse; however, with recent advances in cellular and structural biology, researchers are now performing detailed analyses of the early events in cytokine signaling. The development of novel molecular technologies and multiomics will allow future studies to explore whether STAT-mediated chromatin modification and transcriptional regulation vary in certain stem cell types or microenvironments (that is, characterized by higher specificity and regulation). Finally, stem cells are usually regulated by multiple exogenous, endogenous, and inducible signaling cytokines, and how multiple signaling pathways are coordinated by JAK-STAT remains to be elucidated. Clarifying these uncertainties will provide a theoretical basis for the development of novel therapeutics with higher specificity.

### Others

Transforming growth factor β (TGF-β) is a well-studied cytokine that plays important roles both in the maintenance of normal organismal physiological functions and in the proliferation, migration, metabolic adaptation, and phenotypic plasticity of stem cells.^118^ A recent study employing bioinformatics identified a series of genome-wide long noncoding RNAs (lncRNAs) regulated by the TGF-β signaling pathway. Among these, Gm11549 is a direct target gene of the TGF-β signaling pathway and is specifically highly expressed during the in vitro differentiation of mouse ESCs into the mesodermal cells.^119^ Subsequently, based on biochemical, genetic, cell biology, and metabolomics approaches, we found that Gm11549 encodes a micropeptide known as nodal-enhanced mesendoderm micropeptide (NEMEP) that enhances the glucose uptake of glucose transporter proteins during mesodermal differentiation.^119^ This suggests that the TGF-β signaling pathway can regulate mesoderm development not only from the perspective of modulating the expression of lineage-determining transcription factors but also by regulating cellular glucose metabolism to guide stem cell fate decisions. In addition, intrathecal transporter 20 (IFT20), an IFT complex B proteins, regulates osteoblast differentiation and bone formation; however, how IFT20 regulates the fate of MSCs remains undetermined. IFT20 controls the spectral distribution of MSCs during skeletal development by regulating glucose metabolism through activation of TGF-β-Smad2/3 signaling, enhancing the binding activity of Smad2/3 to the glucose transporter 1 (Glut1) promoter, and upregulating Glut1 expression.^120^

Cellular-myelocytomatosis oncogene (c-Myc) is a multifunctional transcription factor with many targets, which plays an important regulatory role in tissue regeneration and stem cell function. c-Myc is highly responsive to changes in the surrounding environment and maintains the proliferative and differentiation capacities of stem cells by reprogramming their metabolic pathways, including glycolysis, OXPHOS, and lipid metabolism.^121^ This suggests that c-Myc plays an important role in stem cell metabolism. In the adult brain, the transition from quiescence to activation of NSCs is a regulatory focus for neural regeneration, and dysregulation of this transition can lead to brain diseases. One study revealed a central role of c-Myc in the quiescence and homeostasis of NSCs via the coordination of metabolic reprogramming (such as mitochondrial remodeling) with the cell cycle state.^122^ The results of the study also demonstrated how in vitro modeling can be used to gain a comprehensive understanding of how quiescence and activation are coordinated through master cell cycle and metabolic controllers (such as c-Myc).^122^ A systematic understanding of these transitions and their dynamics will help unveil new therapeutic strategies for neural regeneration. Humans long-term HSCs (LT-HSCs) are capable of meeting our daily hematopoietic needs while safeguarding the maintenance of the stem cell banks.^123^ A previous study elucidated the dichotomous regulation of “resting-activation” during early hematopoiesis; when the body senses hematopoietic demand, LT-HSCs increase MYC expression, promote MYC-driven anabolic pathways, competitively inhibit the activity of transcription factor EB (TFEB), inhibit the lysosomal pathway, and prevent the expression of transferrin on the cell membrane surface.^123^ Degradation of transferrin receptor1 (TfR1) on the cell membrane surface promotes the differentiation of LT-HSCs into erythrocytes, whereas TFEB enhances lysosomal activity by promoting the expression of lysosome-related genes, enabling LT-HSCs to maintain their resting state and self-renewal capacity.^123^

Further studies are needed to understand the specific mechanisms by which multiple signaling pathways affect various metabolic pathways in stem cells. Elucidating the interactions between these pathways will allow us to gain a deeper understanding of the complex regulatory networks that control metabolic homeostasis in stem cells.

## Conclusion and prospects

Stem cell metabolic homeostasis is a large and complex field with important application prospects in stem cell therapy and regenerative medicine. First, the study of stem cell metabolic homeostasis can reveal the biological properties and functions of stem cells and provide a theoretical basis and practical guidance for stem cell therapy and regenerative medicine. Second, the study of stem cell metabolic homeostasis can provide a theoretical basis for optimizing stem cell culture and expansion while improving the efficiency and quality of stem cell production. Lastly, the study of stem cell metabolic homeostasis can provide new ideas and methods for stem cell differentiation and regeneration and promote developments in stem cell therapy and regenerative medicine.

Nevertheless, many questions remain unanswered, and future research should be conducted in the following directions:

1. To comprehensively investigate the dynamic changes and adaptive mechanisms of various types of stem cell metabolism.
2. To strengthen our knowledge on the mechanisms of cellular gene regulation and investigate their impact on stem cell metabolism.
3. To determine the differences in the metabolic-regulatory mechanisms of various types of stem cells.
4. To identify whether a widely conserved metabolic mechanism that is unique to stem cells exists and determine whether self-renewal and pluripotency depend on specific aspects of metabolic regulation.
5. To evaluate whether the persistence of stem cells throughout the lifespan of an organism depends on metabolic quiescence, which enforces cell cycle quiescence, and investigate how stem cell division and differentiation affect their self-renewal potential due to the activation of anabolic pathways.
6. To develop novel biological techniques that combine transcriptomics, proteomics, metabolomics, and isotope enrichment profiles with high-resolution histological data for application to stem cells in vivo.

In summary, stem cell metabolism is vital to life, and exploring the involved energy conversion processes and influencing factors is crucial for the advancement of the life sciences. In the future, through in-depth research, we will better understand mechanisms of stem cell metabolism, identify opportunities and challenges in the development of stem cell therapy and regenerative medicine, and make novel contributions to the advancement of biomedicine, bioengineering, and other fields.
